# Phase Angle as a Prognostic Indicator of Survival in Institutionalized Psychogeriatric Patients

**DOI:** 10.3390/nu15092139

**Published:** 2023-04-29

**Authors:** Sara Barrera Ortega, Paz Redondo del Río, Laura Carreño Enciso, Sandra de la Cruz Marcos, María Noel Massia, Beatriz de Mateo Silleras

**Affiliations:** 1 Psycho-Geriatric Area, Assistance Center of San Juan de Dios, 34005 Palencia, Spain; sjdpalencia.bioetica@sjd.es (S.B.O.); mmassia@gmail.com (M.N.M.); 2 Department of Nutrition and Food Science, Faculty of Medicine, University of Valladolid, 47005 Valladolid, Spain; paz.redondo@uva.es (P.R.d.R.); sandra.cruz@uva.es (S.d.l.C.M.); bmateo@uva.es (B.d.M.S.)

**Keywords:** bioelectrical impedance, phase angle, dementia, schizophrenia, psychogeriatric patients, nutritional status assessment

## Abstract

Phase angle (PhA) has been evidenced to be a useful survival indicator and predictor of morbi-mortality in different pathologies, but not in psychogeriatric patients. The aim of this study was to evaluate the clinical utility of PhA as a prognostic indicator of survival in a group of institutionalized psychogeriatric patients. A survival study was conducted on 157 patients (46.5% dementia, 43.9% schizophrenia). Functional impairment stage, frailty, dependence, malnutrition (MNA), comorbidity, polypharmacy, BMI, and waist circumference were registered. Body composition was analyzed using a 50-kHz whole-body BIA; PhA was recorded. The association between mortality and standardized-PhA was evaluated through univariate and multivariate Cox regression models and ROC-curve. The risk of death decreased when Z-PhA, BMI, and MNA were higher. Mortality increases with age, frailty, and dependence. The risk of death was statistically significantly lower (56.5%) in patients with schizophrenia vs. dementia (89%). The Z-PhA cut-off point was −0.81 (Sensitivity:0.75; Specificity:0.60). Mortality risk was multiplied by 1.09 in subjects with a Z-PhA < −0.81, regardless of age, presence of dementia, and BMI. PhA presented a remarkable clinical utility as an independent indicator of survival in psychogeriatric patients. Moreover, it could be useful to detect disease-related malnutrition and to identify subjects eligible for an early clinical approach.

## 1. Introduction

Malnutrition (MN) is a highly prevalent syndrome in older adults. The consequences of MN are severe for geriatric patients since it can decrease functional capacities and increase frailty and morbimortality [1,2,3]. Thus, it is strongly recommended to include the assessment of nutritional status (ANS) in a comprehensive geriatric evaluation. However, despite its usefulness and effectiveness, ANS is often undervalued by health professionals [4]. Due to the lack of a gold standard method to assess the nutritional status of the elderly, it is advisable to do a multidimensional assessment. A combination of various parameters and techniques helps to overcome each of their limitations when considered separately [5].

The inclusion of body composition analysis in the ANS can be very advantageous for the early detection and treatment of MN [6]. Body composition may be determined by different methods, such as magnetic resonance imaging (MRI), computed tomography (CT), bioelectrical impedance analysis (BIA), impedance, ultrasound, or dual X-ray absorptiometry (DXA) [7,8,9,10]. DXA is considered the gold standard method for body composition analysis; however, it is expensive and impractical since its poor portability and the need for highly trained personnel. In this sense, depending on the patients and the available techniques, more operative methods should be used in clinical practice [10]. BIA is a harmless, portable, repeatable, precise, economic, and objective tool that also presents little technical difficulties [6]. Therefore, it is gaining great popularity in daily clinical practice since it is a validated technique for body composition analysis in geriatric patients [3].

It has been documented that alterations in the electrical parameters of BIA are detected earlier than other indicators used for the ANS—anthropometric or biochemical [3,11]. For this reason, BIA would favor an early nutritional approach helping to reduce morbimortality [1,2,3,4] and to improve the vital prognosis in elderly patients. In the context of an exhaustive ANS in geriatric patients, the inclusion of impedance measurement to analyze body composition is undoubtedly very interesting [6,12,13,14,15,16]. Recently, the direct use of the phase angle (PhA), one of the electrical variables, has been proposed not only as an indicator of body composition, but as a prognostic factor [15,16,17].

PhA (relationship between resistance and reactance), obtained by BIA, is an indicator of cell membrane integrity which reflects cell mass. It is considered a good marker of cell function, hydration status, and, therefore, nutritional status [15,18]. Additionally, it has been shown to be useful as (1) a predictor of mortality and risk of complications in different pathologies, (2) a prognostic factor in different types of cancer, and (3) an indicator of survival [4,11,17,19]. An added advantage is that its interpretation is based on its raw value, avoiding dependency on predictive models or any assumptions about morphologies, hydration, etc. [4,18,20]. Currently, studies have evaluated the use of PhA in patients with Alzheimer’s disease (AD) as a nutritional status indicator [21,22] and its association with mortality [22]. However, as far as our concern, there are not any studies that analyze this indicator in psychogeriatric patients with schizophrenia.

The objective of the present study was to evaluate the clinical utility of PhA as a prognostic indicator of survival in a group of institutionalized psychogeriatric patients.

## 2. Materials and Methods

A survival study was conducted in a group of institutionalized subjects from a psychogeriatric center in Palencia (Spain). At the moment of the evaluation (October 2010), they did not present any disease nor contraindications for BIA (disturbances of water and electrolyte balance, amputations, metal implants or implanted cardiac devices). Survival data were collected until June 2021. The study was conducted according to the guidelines of the Declaration of Helsinki, and written informed consent was obtained from all volunteers or their legal representatives. Approval was obtained from the Clinical Research Ethics Committee (CEIC) of Valladolid-East Health Area (Protocol code: PI 14-215).

Clinical and demographic data were registered from medical records (sex, age, length of stay, disease, and medication). According to the Diagnostic and Statistical Manual of Mental Disorders, Fifth Edition (DSM-5) [23] and the International Classification of Diseases, 10th Revision (CIE-10) [24] criteria, subjects were classified as dementia patients (DP), schizophrenia patients (SP), and other psychiatric pathologies patients (OPP). The stage of functional deterioration of dementia patients was determined with the FAST scale [25]. FAST greater than 7c was established as a highly evolved stage with palliative needs [26]. Frailty was assessed with the FRAIL test [27] and dependency using the Barthel index [28]. All subjects underwent a complete Mini Nutritional Assessment (MNA) [29]. Comorbidity was assessed with Charlson Comorbidity Index (CCI); high comorbidity was considered if the CCI score was five or higher [30]. Polypharmacy was defined as the concurrent use of five and more drugs [30].

Anthropometric measurements (weight, height or heel-knee distance, and body perimeters) were determined following the NHANES [31] and WHO [32] protocols by using conventional methods (SECA Hamburg scale and vertical stadiometer, and Cescorf non-extensible tape measure). The nutritional assessment was established following the protocol of the Spanish Society of Parenteral and Enteral Nutrition (SENPE) and the Spanish Society of Geriatrics and Gerontology (SEGG) in their consensus document for geriatric patients [33]. BMI was cataloged with the WHO cut-off points [34] and the consensus document for the elderly population [32]. Waist circumference was evaluated with the WHO cut-off points [35].

Whole-body BIA was performed with a tetrapolar electrode configuration in monofrequency mode at 50 kHz with a BIA-101 analyzer (AKERN-Srl, Florence, Italy), following the standard protocol of Lukaski [36]. Raw electrical variables [resistance (R), reactance (Xc), and phase angle (PhA)] were collected, and body compartments were estimated: fat-free mass (FFM) using the Kyle et al. model [37]; and skeletal muscle mass (SMM), using the Janssen model [38]. Normalized Z-scores of body composition variables and PhA were calculated by using the reference values for the European Caucasian population published by Kyle et al. [37].

Categorical variables are described as absolute (*n*) and relative (%) frequencies, and quantitative variables as mean (SD). Differences between the quantitative variables were analyzed with Student t or ANOVA tests with Scheffé post hoc test for independent measurements. Differences in categorical variables were evaluated with the Chi-square test.

A Cox proportional hazards analysis was performed to estimate the hazard ratio (HR) and 95% confidence interval (95% CI) of PhA, age and other variables related to nutritional status (BMI, MNA), functional (FRAIL, Barthel categories) and main pathology (dementia, schizophrenia, or other psychiatric pathologies) for survival time. A ROC curve was constructed, and the area under the curve (AUC) was determined to assess the discriminatory capacity of the Z-PhA (quantitative variable) for survival time (categorical variable exitus). The optimal cut-off point for the variable was established based on the best combination of sensitivity and specificity. Subsequently, multivariant Cox regression models were adjusted to calculate the HR (95% CI) for Z-PhA (as a quantitative variable and as a categorical variable, based on the previously established cut-off point) with the variables that were statistically significant in the univariate analysis for survival time (forward stepwise method (conditional)). Statistical significance was set at *p* < 0.05. Statistical analysis was performed with the statistical package SPSS 19.0 for Windows.

## 3. Results

### 3.1. Participants’ Characteristics

The sample was formed by 158 elderly subjects institutionalized in two units of a psychogeriatric center in Palencia (Spain), 106 men (67.1%) and 52 women (32.9%), with a mean age of 76.9 years (8.9). The final sample size was 157 subjects due to the transfer of a woman to another center during the study follow-up. Table 1 shows the clinical and demographic characteristics of the subjects. Most of SP (92.8%) and OPP (66.7%) were men, while women predominated in the DP group (men: 43.8%). Regarding the FAST scale, 69.9% (51 patients) of DP were classified as FAST ≤ 6; 9.6% (7 patients) were classified as FAST 7a and 7b; and the remaining 20.5% (15 patients) as FAST ≥ 7c.

### 3.2. Nutritional Status and Body Composition

Table 2 lists the classification of subjects according to the nutritional status indicators studied. The mean MNA score was 18.4 points (3.6), and the mean BMI was 24.5 kg/m^2^ (4.1). MNA detected that more than half of the sample presented malnutrition or risk of malnutrition (53.5%), whereas BMI registered a much lower value (27.4%). Additionally, BMI showed that the fourth part of the sample had some excess of weight. Finally, waist circumference indicated that the third part of the subjects presented a risk of metabolic complications. Patients who presented dementia registered statistically significantly lower punctuation in MNA, BMI, and waist circumference than the others.

Table 3 summarizes body composition data estimated by BIA. Normalized body compartment scores reflected maintenance or increase of fat mass (FM) and depletion of fat-free mass (FFM) and muscle mass. Dementia patients registered statistically significantly lower fat mass index (FMI) than the others.

Raw electrical values of BIA in the sample were: R = 576.7 Ohm (90.6) (552.4 Ohm (77.4) and 627.1 Ohm (96.0) in men and women, respectively), Xc = 43.2 Ohm (9.3) (44.3 Ohm (8.9) and 40.7 Ohm (9.7) in men and women, respectively), and PhA = 4.27 degrees (0.95) (4.55° (0.84) and 3.69° (0.92) in men and women, respectively). The mean Z-PhA value was −0.809 (0.9). In the DP group, this value (−1.15 (0.8)) was statistically significantly lower than the rest of the groups: SP (−0.51 (0.9)) and OPP (−0.54 (0.9)).

### 3.3. Phase Angle, Functional and Nutritional Status, and Mortality Risk

After 10 years of follow-up, 113 residents (72%) were deceased (65 DP (89.0%), 39 SP (56.5%), and 9 OPP (60.0%)). These subjects showed statistically significantly lower Z-PhA and BMI. Additionally, they were older than patients who were alive at the end of follow-up (Table 4). A higher risk of death was observed in patients with (1) frailty or risk of frailty, (2) moderate, severe, and total dependency, and (3) malnutrition risk or undernutrition (Table 4).

Mean survival time was 59.6 months (44.5) (median: 53 months; range: 0 to 131). Table 5 shows Cox univariate regression analysis, which corroborates the previous results. Thus, in the sample studied, the risk of death decreased when Z-PhA, BMI, and MNA were higher. Mortality increases with age, frailty (higher FRAIL punctuation), and dependence (lower Barthel index punctuation). On the other hand, the risk of death was statistically significantly reduced by 68% in patients with schizophrenia compared to patients with dementia.

**Figure 1:**
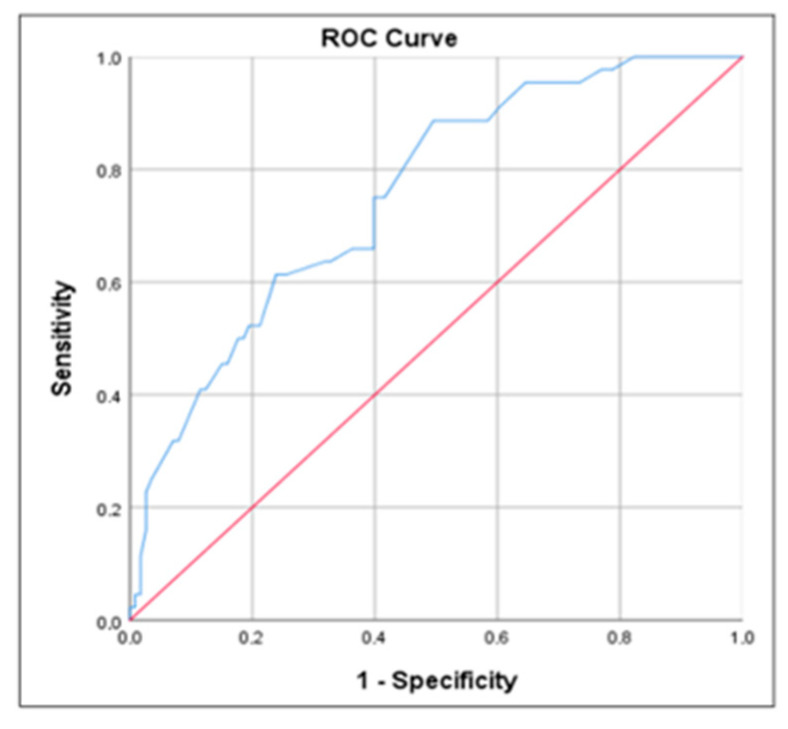
ROC curve of the standardized phase angle to detect survival time.

The mean Z-PhA value was −0.809 (0.90). The area under the ROC curve (Figure 1) was 0.75 (95% CI: 0.67–0.83; *p* < 0.001). The Z-PhA cut-off point was −0.81 (Sensitivity: 0.75; Specificity: 0.60).

The Z-PhA variable was categorized by using the ROC curve cut-off point obtained (Z-PhA ≤ −0.81 vs. Z-PhA > −0.81). Finally, the multivariant Cox regression model built determined that mortality risk was multiplied by 1.9 in subjects with a Z-PhA lower than −0.81, regardless of age, presence of dementia, and BMI (Table 6).

## 4. Discussion

The present study has been carried out in a group of elderly patients institutionalized in a psychogeriatric center. The group was classified according to the main clinical diagnosis that subjects presented: dementia (46.5%) or schizophrenia (43.9%). In our sample, the number of men doubled the number of women, which is very uncommon compared to the general geriatric population. The study was conducted in two different units of the same healthcare center, and one of them formerly used to be an only men’s psychiatric center. Even though patients of both sexes have been admitted for years, there continues to be a predominance of men who were admitted at a young age and have grown old in the center. That is also the reason why there was a high number of men with schizophrenia diagnosis (92.8%).

Our results evidenced that patients with dementia presented a higher deterioration of their health status than the rest of the subjects: higher frailty, increased level of dependence, worse nutritional status, and higher comorbidity. The nutritional status of most of the SP group was normal (79.9% according to MNA; 46.4% according to BMI), with a mild level of dependency (85.5%), and 58% of them presented a risk of frailty. Additionally, they registered statistically significantly lower comorbidity than the DP group (42% vs. 89%). Since (1) hospitalization enabled a better monitorization of pathologies and control of comorbidity, and (2) an elderly age is related to a higher functional impairment, these results could be explained by the younger age and the higher length of stay in the center of SP group (SP: 34 years; DP: 10.8 years).

More than half of the subjects (53.5%) presented malnutrition or risk of malnutrition, according to MNA. BMI only detected malnutrition or risk of malnutrition in 27.4% of the subjects. In elderly patients, it has been observed that normal or even high BMIs can mask situations of malnutrition, and weight can increase at the expense of fat mass or edema [5]. In fact, a quarter of the sample was overweight, and a third was at risk of metabolic complications based on waist circumference. Since various authors have documented that BMI is not a good marker of nutritional status in the elderly population [5], it is recommended to complete the nutritional status assessment with a study of body composition [39]. The analysis of the body composition of our sample showed a statistically significant reduction in FFM and skeletal muscle mass with excessive or normal FM (Table 2). Previous studies have reported the same results in patients with dementia, usually with Alzheimer’s disease (AD) [40,41,42], which is common in the elderly population of our environment, as described in other studies [43,44]. In the present study, SP and OPP presented a statistically significantly higher FM than DP. This situation could be aggravated by an increased cardiometabolic risk (waist circumference). Metabolic abnormalities are especially prevalent in severe mental illness patients. Independently of medical causes, unhealthy lifestyles, and disparities in health care contribute to this high prevalence. Additionally, the use of psychotropic medication, such as antipsychotics, for example, can increase the risk of metabolic abnormalities in these patients [45].

During follow-up, 72% of subjects passed away. Mortality was significantly higher in the most vulnerable patients: older, with greater dependency, frailty or risk of frailty, malnutrition or risk of malnutrition, more advanced stage of dementia (FAST ≥ 7) and lower BMI and FM (Table 4). This explains the short mean survival time observed, less than 5 years. In addition, mortality was higher in DP than in SP. De Sousa et al. previously documented a higher risk of mortality in patients with AD who presented a worse nutritional and functional status, regardless of sex, age, and mental function [22]. Dementia itself should be considered a risk factor for malnutrition [46], causing increased morbidity and mortality compared to other processes [39]. It was also evidenced that the risk of mortality in patients with higher BMI and FM was lower, which was demonstrated in other studies with DP as well [22,47].

Our results highlighted that deceased patients had a significantly lower Z-PhA than alive patients, as De Sousa et al. also showed in community-dwelling older adults with AD [22]. Z-PhA value in DP was statistically significantly lower than in SP (−1.15 vs. −0.51). As it has previously mentioned, DP presented more frailty, age, level of dependency, comorbidity, and a worse nutritional status, which could explain their higher risk of mortality and lower PhA.

Different studies documented that PhA decreases with age and is greater in men than in women (except in those older than 70–80 years). It also depends on ethnicity, the presence of different pathologies, and, in the case of the elderly population, institutionalization [11,15,20]. Reference values for PhA have been developed for different populations, ages, ethnic groups and even BMI [37,48,49,50,51,52,53]. A recent systematic review of 46 studies (249, 844 subjects) established PhA reference values for populations from 70 to 80 years (men: 5.3° (95% CI: 4.5–6.0); women: 5.4° (95% CI: 5.3–5.6)) and over 80 years (men: 5.6° (95% CI: 4.8–6.4); women: 5.1° (95% CI: 4.7–5.5)) [50]. The mean PhA value in our sample was much lower than these reference values (4.55° in men and 3.69° in women). However, earlier studies have also shown different PhA values in patients with AD by reporting both higher [21,41,42,54] and lower [22] values than our sample. Given the differences in electrical variables according to the measurement protocol and equipment used, the data should only be compared with studies using similar methodology [55,56].

Recently, a systematic review analyzed the utility of PhA as an indicator of mortality in different clinical situations. Their sample included patients with illnesses, such as kidney disease, heart disease, critically ill patients, sclerosis, liver disease, pulmonary disease, cancer, or HIV, but not psychiatric diseases [57]. The ROC curve built to validate the Z-PhA as an indicator of survival in our sample showed that a Z-PhA cut-off point of −0.81 registered adequate values for sensitivity (0.75) and specificity (0.60), with an area under the curve of 0.75 (95% CI: 0.67–0.83). In comparison to prior studies, it must be pointed out that this value is much lower than observed by Genton et al., in Swiss patients over 65y, with different pathologies [55], and the documented ones as reference values in previous populations [11,14,20,48,49,57]. Several studies have established the PhA cut-off point as an indicator of mortality in older adults, for example, the group of Kwon et al. (value of 3.19°) [58]. However, their result was obtained by using the PhA value for the non-stratified sample by sex, when the most appropriate would have been standardizing PhA to avoid the effect of sex since it has been demonstrated that PhA is lower in women than in men [50]. The PhA cut-off point as an indicator of mortality obtained in our sample of psychogeriatric patients (without standardization nor stratification by sex) was 4.25°, which is substantially higher than the one reported by Kwon et al. [58]. These results could be explained by differences in the studied subjects: our sample was mainly composed of Caucasian men (67.1%), while 75.6% of the Kwon sample was formed by very elderly Korean women, which could clarify the reduced PhA value reported by those authors. To date, there is no reference cut-off value for PhA or Z-PhA because it depends on population, illness, nutritional status, measurement protocol and BIA device [56].

The PhA as a prognostic indicator of survival was maintained after correcting the effect of age, BMI, dependency, and dementia diagnosis. In fact, the multivariant Cox regression model in our study confirmed that the risk of mortality was almost double in subjects with a Z-PhA less than −0.81, regardless of BMI, age, dependency status, or psychiatric pathology. A Z-PhA cut-off value of −1.41, as an indicator of risk of mortality, was reported by Genton et al. in elderly subjects with different illnesses [55]; however, the BIA device they employed was not the same equipment as our research group. The association between PhA and mortality has been shown only when using certain BIA devices (as the one employed in this study) but not with all of them [59]. Several studies have also demonstrated this association specifically in older patients, regardless of age, sex, comorbidities, and BMI categories, but they did not analyze psychogeriatric patients [17,55,58,59].

Therefore, evidence suggests that PhA is an indicator highly predictive of mortality risk, although its specific individual association with the geriatric diseases and syndromes (and the nutritional and functional status associated with them), so frequent in older adults, should be investigated.

### Limitations of This Study

The psychogeriatric patients in this study have been institutionalized for a very long time, and, in addition, they presented serious comorbidities and chronic pathologies, which are higher than the registered ones in non-institutionalized patients of similar age [60]. As a result, our sample presented a high generalized deterioration. Due to the fact the study took place in a psychogeriatric center, it is impossible to compare our sample with patients of similar age, who live at home, and with active aging.

## 5. Conclusions

The usefulness of the PhA as an indicator of survival in elderly psychogeriatric patients has been supported by this study. It also provides a specific cut-off point for the Z-PhA with a higher risk of mortality, regardless of functional and nutritional status. The PhA determination as a routine in elderly patients would permit us to propose an early approach for malnutrition in a quick and simple way. Consequently, morbidity and mortality, functional deterioration, and hospitalizations of these patients would be reduced, favoring their quality and quantity of life.

A new line of research in elderly subjects with dementia has been started with this research. In order to continue with this field of study, it would be interesting to assess the PhA values in non-institutionalized patients. This would allow a more detailed analysis of the effect of the greater deterioration that justifies institutionalization. Additionally, it would be interesting to conduct longitudinal studies to analyze the probable association of PhA with the different pathologies and geriatric syndromes as they arise in older patients.

## Figures and Tables

**Table 1:** Clinical and demographic characteristics of the patients.

Variables		All	Dementia	Schizophrenia	OPP
*n* (%)		157 (100)	73 (46.5)	69 (43.9)	15 (9.6)
Age (y.) (mean (SD))		76.9 (8.9)	80.3 (9.5) ^#^	74.3 (7.0)	72.5 (8.2)
Length of stay (y.) (mean (SD))		21.5 (19.8)	10.8 (12.9)	34 (19.6) *	16.2 (15.6)
FRAIL test (*n* (%))	Frailty	70 (44.6)	52 (71.2) ^#^	15 (21.7)	3 (20.0)
	Risk of frailty	64 (40.8)	19 (26.0)	40 (58.0) *	5 (33.3)
	Non frailty	23 (14.6)	2 (2.7)	14 (20.3)	7 (46.7)
Barthel test (*n* (%))	Mild dependency	85 (54.1)	15 (20.5)	59 (85.5) *	11 (73.3)
	Moderate dependency	50 (31.8)	40 (54.8) ^#^	7 (10.1)	3 (20.0)
	Severe dependency	7 (4.5)	4 (5.5)	2 (2.9)	1 (6.7)
	Total dependency	15 (9.6)	14 (19.2) ^#^	1 (1.4)	0 (0)
High comorbidity (CCI) (*n* (%))		96 (61.1)	65 (89.0) ^#^	29 (42.0)	2 (13.3)
Polypharmacy (*n* (%))		142 (90.4)	71 (97.3) ^&^	58 (84.1)	13 (86.7)

OPP: other psychiatric pathologies; CCI: Charlson Comorbidity Index; ^#^ *p* < 0.001 for comparison dementia patients vs. the rest; ^&^ *p* = 0.024 for comparison dementia patients vs. the rest; * *p* < 0.001 for comparison schizophrenia patients vs. the rest.

**Table 2:** Classification of the participants in the study according to some indicators of nutritional status.

Indicator	Variable/Category	Psychiatric Pathology N (%) If No Other Is Indicated		
		Dementia	Schizophrenia	OPP
MNA	MNA (points) (mean (SD))	17.8 (3.3) *	21.6 (3.7)	20.0 (4.4)
	MN	22 (30.1)	3 (4.3)	1 (6.7)
	Risk of MN	42 (57.5)	11 (15.9)	5 (33.3)
	Normal nutritional status	9 (12.3)	55 (79.9)	9 (60.0)
BMI	BMI (kg/m^2^) (mean (SD))	23.4 (4.2) *	25.2 (3.8)	26.5 (3.8)
	MN (BMI < 18.5 kg/m^2^)	9 (12.3)	1 (1.4)	0 (0)
	Risk of MN (BMI: 18.5–21.9 kg/m^2^)	16 (21.9)	15 (21.7)	2 (13.3)
	Normal (BMI: 22–26.9 kg/m^2^)	37 (50.7)	32 (46.4)	6 (40.0)
	Overweight (BMI: 27–29.9 kg/m^2^)	1 (1.4)	14 (20.3)	3 (20.0)
	Obesity (BMI: 30 kg/m^2^)	10 (13.7)	7 (10.1)	4 (26.7)
Waist circumference	Risk of metabolic complications	24 (33.8)	21 (31.8)	6 (40.0)
	No risk	47 (66.2)	45 (68.2)	9 (60.0)

OPP: other psychiatric pathologies; MNA: mini-nutritional assessment; BMI: Body mass index; MN: malnutrition. * *p* < 0.05 for comparison between dementia vs. schizophrenia and other psychiatric pathologies.

**Table 3:** Standardized body composition variables of the participants in the study.

Body Composition Variables (Mean (SD))	All Subjects	Groups		
		Dementia	Schizophrenia	OPP
Z-FFMI	−0.95 (1.2)	−1.11 (1.1)	−0.87 (1.2)	−0.56 (1.3)
Z-FMI	0.39 (1.2)	0.03 (1.1) *	0.64 (1.1)	0.99 (1.4)
Z-SMMI	−0.87 (0.97)	−0.90 (0.82)	−0.90 (1.1)	−0.58 (1.2)

OPP: other psychiatric pathologies; FFMI: fat-free mass index; FMI: fat mass index; SMMI: skeletal muscle mass index. * *p* < 0.05 for comparison between dementia vs. schizophrenia and other psychiatric pathologies.

**Table 4:** Characteristics description of subjects according to survival.

Variables		Exitus	
		No (*n* = 44)	Yes (*n* = 113)
Age (years) (mean (SD))		71.5 (6.6)	79.1 (8.8) *
BMI (kg/m^2^) (mean (SD))		26.1 (3.6)	23.9 (4.1) *
R (Ohm) (mean (SD))		550.3 (86.5)	587.0 (90.5) *
Xc (Ohm) (mean (SD))		48.2 (8.9)	41.2 (8.7) *
Z-PhA (mean (SD))		−0.24 (0.79)	−1.03 (0.84) *
MNA (points) (mean (SD))		20.9 (4.0)	17.8 (3.3) *
Nutritional status (*n* (%))	Undernutrition	3 (11.5)	23 (88.5) ^#^
	Malnutrition risk	8 (13.8)	50 (86.2) ^#^
	Normal	33 (45.2)	40 (54.8)
Frailty (*n* (%))	Frailty	11 (15.7)	59 (84.3) ^#^
	Frailty risk	20 (31.3)	44 (68.8) ^#^
	Non frailty	13 (56.5)	10 (43.5)
Dependency (*n* (%))	Total	0 (0)	15 (100) ^#^
	Severe	0 (0)	7 (100) ^#^
	Moderade	6 (12.0)	44 (88) ^#^
	Mild	38 (44.7)	47 (55.3)

BMI: body mass index; R: resistance; Xc: reactance; Z-PhA: standardized phase angle. * *p* < 0.05 for comparison between exitus vs. non-exitus. ^#^ *p* < 0.001 for comparison between exitus vs. non-exitus between nutritional status, frailty and dependence categories.

**Table 5:** Cox proportional hazards univariate regression analysis of the relationship between different variables and survival time.

Variable		HR (Survival)	95% CI	*p*
Z-Phase angle		0.47	0.36–0.60	<0.001
Age		1.07	1.04–1.09	<0.001
Pathology	Dementia	1.00		<0.001
	Schizophrenia	0.32	0.21–0.47	<0.001
	Others	0.30	0.15–0.64	0.002
FRAIL	Normal	1.00		<0.001
	Fragility risk	2.29	1.12–4.71	0.024
	Fragility	4.69	2.31–9.54	<0.001
Barthel	Low dependence	1.00		<0.001
	Moderate dependence	2.92	1.92–4.44	<0.001
	High dependence	14.67	6.19–34.75	<0.001
	Total dependence	5.25	2.90–9.51	<0.001
BMI		0.90	0.85–0.95	<0.001
MNA		0.91	0.84–0.97	0.006

HR: hazard ratio; CI: confidence interval; BMI: Body mass index; MNA: mini-nutritional assessment.

**Table 6:** Multivariant Cox regression analysis of the relationship between different variables and survival time.

Variable		HR (Survival)	95% CI	*p*
Z-Phase angle (≤−0.81 vs. >−0.81)		1.90	1.27–2.85	0.002
Age		1.04	1.01–1.06	0.005
Pathology	Dementia	1.00		0.001
	Schizophrenia	0.45	0.29–0.70	<0.001
	Others	0.48	0.22–1.03	0.061
BMI		0.94	0.89–0.99	0.011

HR: hazard ratio; CI: confidence interval; BMI: Body Mass Index.

## Data Availability

Not applicable.
